# *Bauhinia variegata candida* Fraction Induces Tumor Cell Death by Activation of Caspase-3, RIP, and TNF-R1 and Inhibits Cell Migration and Invasion* In Vitro*

**DOI:** 10.1155/2018/4702481

**Published:** 2018-03-25

**Authors:** K. M. Santos, I. N. F. Gomes, R. J. Silva-Oliveira, F. E. Pinto, B. G. Oliveira, R. C. R. Chagas, W. Romão, R. M. V. Reis, R. I. M. A. Ribeiro

**Affiliations:** ^1^Laboratory of Experimental Pathology, Federal University of São João del-Rei (UFSJ), West Center Campus Dona Lindu, 35.501-296 Divinópolis, MG, Brazil; ^2^Molecular Oncology Research Center (CPOM), Barretos Cancer Hospital, 14.784-400 Barretos, SP, Brazil; ^3^Petroleomic and Forensic Chemistry Laboratory, Department of Chemistry, Federal University of Espírito Santo (UFES), 29075-910 Vitória, ES, Brazil; ^4^Analytical Chemistry Laboratory, Federal University of São João del-Rei (UFSJ), West Center Campus Dona Lindu, 35.501-296 Divinópolis, MG, Brazil; ^5^Life and Health Sciences Research Institute (ICVS), Health Sciences School, University of Minho, Braga, Portugal; ^6^ICVS/3B's-PT Government Associate Laboratory, Guimarães, Braga, Portugal

## Abstract

Metastasis remains the most common cause of death in cancer patients. Inhibition of metalloproteinases (MMPs) is an interesting approach to cancer therapy because of their role in the degradation of extracellular matrix (ECM), cell-cell, and cell-ECM interactions, modulating key events in cell migration and invasion. Herein, we show the cytotoxic and antimetastatic effects of the third fraction (FR3) from* Bauhinia variegata candida* (Bvc) stem on human cervical tumor cells (HeLa) and human peripheral blood mononuclear cells (PBMCs). FR3 inhibited MMP-2 and MMP-9 activity, indicated by zymogram. This fraction was cytotoxic to HeLa cells and noncytotoxic to PBMCs and decreased HeLa cell migration and invasion. FR3 is believed to stimulate extrinsic apoptosis together with necroptosis, assessed by western blotting. FR3 inhibited MMP-2 activity in the HeLa supernatant, differently from the control. The atomic mass spectrometry (ESI-MS) characterization suggested the presence of glucopyranosides, D-pinitol, fatty acids, and phenolic acid. These findings provide insight suggesting that FR3 contains components with potential tumor-selective cytotoxic action in addition to the action on the migration of tumor cells, which may be due to inhibition of MMPs.

## 1. Introduction

Cervical cancer (CC) is the fourth most frequent type of neoplasm among women in the worlds and the main cause of cancer-related death in low- and middle-income countries [1]. In 2012, more than 270,000 CC deaths occurred worldwide [2]. Human papilloma virus (HPV) is the main etiologic infectious agent associated with cervical cancer. 70% of cases of cervical cancer are induced by HPV types 16 and 18, but over 200 types of HPV have been identified [3]. HPV interferes with the cell cycle regulation because it infects the cervical mucosa and integrates its genome in cells. The viral E6 and E7 oncoproteins are critical for inducing malignant transformation because they lead to the suppression of p53 and pRB, fundamental tumor suppressor genes. Persistent infection leads to cervical intraepithelial neoplasia (CIN), which, if untreated, may progress to cancer cervical [4].

Metastasis is the main cause of cancer death [5]. One of the first steps in the metastatic cascade includes the intravasation of tumor cells into the circulation, and the processes correlated with this event, such as migration and cell invasion, are dependent on the tumor microenvironment [6]. Cytoskeletal rearrangements, combined with the degradation of cell-cell and cell-extracellular matrix (ECM) interactions, are the main mechanisms of premetastatic tissue remodeling [7]. ECM metalloproteinases (MMPs), especially MMP-2 and MMP-9, cleave ECM components, degrade the basement membrane, and allow tumor cells to penetrate the adjacent matrix stroma [8]. This type of remodeling has been associated with invasive CCs [9] and overexpression of MMP-2 and MMP-9 is also correlated with a worse prognosis in patients with this tumor type [10].

The lack of improvement in cervical cancer treatment is mainly due to resistance to cytotoxic agents [11]. Thus, the search for new therapies with low cost and minimal side effects has become paramount, with many researchers looking for compounds with anticancer activity from natural compounds [12]. Recent studies by our research group have shown that* Bauhinia ungulata* extract fractions have potential antimetastatic activity [13].

In the present work, we characterized phytochemically a* B. variegata candida* fraction with inhibitory activity against MMPs and established the mechanism of action of these compounds on cell viability in human cervical carcinoma (HeLa) and human peripheral blood mononuclear cells (PBMCs). Migration and invasion, MMP-2 activity, and the cell death pathways of HeLa cells treated with this fraction were also evaluated.

## 2. Materials and Methods

### 2.1. Sample and Preparation of Extracts

*Bauhinia variegata candida* (Bvc) stems were collected from an urban area of Divinópolis, Brazil, 20°08′20′′S; 44°53′02′′W; 712 m, and powdered. The powdered material (100 g) was extracted with 70% ethanol (600 mL, five times) for seven days at room temperature to obtain the crude extract. The crude extract was partitioned using hexane, ethyl acetate, and chloroform (160 mL, each). From this, the ethyl acetate partition (PA) was fractionated by open chromatographic column (silica gel 60 G). The column was eluted with hexane, dichloromethane, ethyl acetate, methanol, and water (50/50, v/v), obtaining seven fractions. All extracts were frozen (−80°C) and lyophilized.

### 2.2. Cancer Cell Line, Cell Culture Conditions, and Antibodies

Human cervical adenocarcinoma cells (HeLa) were maintained in Dulbecco's Modified Eagle Medium (DMEM) supplemented with 10% (v/v) heat inactivated fetal bovine serum (SBF) and 1% streptomycin/penicillin (v/v) and maintained in a humidified atmosphere of 5% CO_2_ at 37°C [14]. The commercial cell line HeLa was authenticated by STR analysis and tested for mycoplasma contamination by PCR [15]. The DMEM culture medium, (3-(4,5-Dimethylthiazol-2-yl)-2,5-Diphenyltetrazolium Bromide) (MTT), Ficoll-Paque Plus, and MMP-2 and MMP-9 (M9445 and M8945, resp.) were purchased from Sigma Aldrich (St. Louis, Missouri, USA). Fetal bovine serum and Bioacoats were purchased from Becton Dickinson (BD) (Bedford, MA, USA). Antibodies were purchased from Cell Signaling Technology (Danvers, Massachusetts, USA).

### 2.3. Peripheral Blood Mononuclear Cells

Peripheral blood mononuclear cells (PBMCs) were isolated from fresh blood (40 mL) obtained by venipuncture from two healthy volunteers. The obtained blood was centrifuged by density gradient using Ficoll-Paque Plus. The isolated cells were suspended in DMEM culture medium (10% FBS and 1% v/v). Adherent cells were considered mononuclear leukocytes [16]. This work was approved by the Ethics Committee in Research with Human Beings of the Federal University of São João del-Rei, under the number of opinions: 2.007.582.

### 2.4. Zymography

Each fraction of* B. variegata candida* was diluted in dimethyl sulfoxide (DMSO 1%) and applied 90 *μ*g/mL in each well to 10% SDS-polyacrylamide gel containing 1 mg/ml gelatin, along with 1.8 *μ*g/mL of MMP-2 and MMP-9 diluted in sample buffer (2.5% SDS and 1 g% sucrose). Electrophoresis was performed under reducing conditions according to [13]. Photographs of the gels were obtained and the MMPs activities, indicated by clear bands, were quantified by densitometry (ImageJ1.42q/Java1.6.0-10). The fraction capable of totally inhibiting the gelatinolytic activity of MMP-2 and MMP-9 was directed to subsequent tests.

For analysis of active MMP-2 in the cell culture supernatant, 5 × 10^5^ HeLa cells/well were plated in a 24-well plate and incubated for 24 hours. The cells were treated with DMEM (2% FBS) with or without the selected fraction (IC_50_ concentration). After 24 hours, the cell culture medium was collected, lyophilized, and diluted in sample buffer (2 : 1, v/v). In each well, 20 *μ*g/proteins were applied and electrophoresis conducted under the same conditions.

### 2.5. Cell Viability Assay

Cell viability was determined by MTT assay. Hela and PBMCs cells were plated in 96-well plates (2.5 × 10^5^ cells/100 *μ*l/well) and incubated in 5% CO_2_, at 37°C. After incubation for 24h the medium was removed, and cells were treated with 5, 10, 25, and 50 *μ*g/ml of the fraction for 72 h. Then, 100 *μ*L of DMEM with MTT (2.5 mg/mL) was added to each well and cells were incubated at 37°C for 3 h. After this time, the formazan crystals formed was dissolved by adding 100 *μ*l/well of DMSO and the optical density was measured using microplate reader (Power Wave XS2 Biotek) at 570 nm.

### 2.6. Wound Healing Assay

The HeLa cells were seeded (5 × 10^5^ cells/well) in 24-well plates and left for 24 h, at 37°C in CO^2^ (5%) until 90% confluency [17]. Then, the adherent cell layer was scratched with a sterile tip of pipette 200 *μ*l. Cell debris was removed by washing with PBS and the cells were treated with the selected fraction (15 *μ*g/mL). Photographs of the wounds were obtained at 0 and 72 hours of treatment using a microscope Axio Vert A1 FL (Carl Zeiss®), at 400x magnification, and the relative migration distance was measured by the following formula:

(1) $$\begin{array}{c} \text{percentage of wound closure}\left(\;\%\;\right)=\frac{100\times \left(A-B\right)}{A}, \end{array}$$

where *A* corresponds to the width of cell wounds before incubation and *B* the width of cell wounds after incubation [18].

### 2.7. Invasion and Migration Assay

Hela cells (2.5 × 10^4^) were plated on the chambers Matrigel coated transwell (8 mm pore-size) in 24-well plates. Inside the chambers 1 mL of DMEM (serum free) containing 15 and 25 *μ*g/mL of the treatment was added whereas DMEM containing 10% SFB was added outside of the chamber. Cisplatin (15 *μ*g/mL) was used as control [19]. After 24 hours, cells that had degraded the Matrigel and invaded to the lower surface of the Matrigel coated membrane were fixed (methanol 70%) and stained with Hematoxylin/Eosin. The cells were photographed (50x) and counted. The same procedure was performed using the plates without Matrigel to evaluate the migratory capacity of the cells [15].

### 2.8. Western Blot Analysis

HeLa cells were treated for 24 hours at the concentrations of 15, 25, and 40 *μ*g/ml of FR3. The dose choice was based on the IC_50_ value calculation. After that, the cells were lysed in a buffer containing 50 mM Tris pH 7, 150 mM NaCl, 5 mM EDTA, 1 mM Na_3_VO_4_, 10 mM NaF, 10 mM sodium pyrophosphate, 1% NP-40, and Protease Inhibitors containing 1 mM Na_3_VO_4_, 10 mM NaF, 10 mM sodium pyrophosphate, 1% NP-40, 10 *μ*g/ml leupeptin, 10 *μ*g/ml aprotinin, 1 mM DTT, 0.01 M EDTA, and 1 mM PMSF. After electrophoresis, the proteins from the gel were transferred (TE 70 PWR Semi-Dry Transfer Unit, GE Healthcare) to a nitrocellulose membrane (Amersham Protram supported 0.45 *μ*m NC, GE Healthcare) using a buffer with 25 mM Tris, 193 mM glycine, and 20% methanol. The expressions of the total and cleaved Parp (# 4967), total and cleaved caspase-8 (# 9746), total and cleaved caspase-3 (# 9662), FAS protein (# 4233), RIP protein (# 4926), and tumor necrosis receptor 1 (TNF-R1) (# 3736) were evaluated. All antibodies were diluted and incubated, according to the manufacturer's recommendations (1/1000 v/v). After washing with TBS-T, membranes were incubated with the secondary anti-rabbit antibody (# 7074) at 1 : 5000 (v/v) dilution. Then, the strip detection was performed by chemiluminescence (ECL, Western Blotting Detection Reagents, RPN2109; GE Healthcare, Piscataway, NJ) using a 1 : 1 (v/v) dilution and subsequently the membranes were photographed using ImageQuant LAS 4000 mini GE Healthcare). All experiments were performed in triplicate.

### 2.9. ESI(−)FT-ICR MS

Fourier transform ion cyclotron resonance mass spectrometry (FT-ICR MS) analysis was performed using a mass spectrometer (model 9.4 T Solarix, BrukerDaltonik GmbH, Bremen, Germany). The extract FR3 was analyzed by negative electrospray ionization, ESI(−), which was infused at a flow rate of 4 *μ*L min^−1^ into the ESI source. The ESI(−) source conditions were set as follows: nebulizer gas pressure of 1.5 bar, capillary voltage of 3.9 kV, transfer capillary temperature of 200°C, and endplate offset of −500 V. The front and back trapping voltages in the ICR cell were +0.80 V and +0.85 V, respectively. The ion accumulation time in the hexapole was 0.010 s, followed by transport to the analyzer cell (ICR) through the multipole ion guide system (another hexapole). The mass spectra were acquired by accumulating 64 scans of time-domain transient signals in four mega-point time-domain data sets. The mass spectrum was processed using the Compass Data Analysis software package (BrukerDaltonics, Bremen, Germany). The proposed molecules were assigned using the Chemspider database (http://www.chemspider.com). The degree of unsaturation for each molecule was deduced directly from its DBE value according to equation: DBE = *c* − *h*/2 + *n*/2 + 1, where *c*, *h*, and *n* are the carbon, hydrogen, and nitrogen numbers, respectively, in the molecular formula [20].

### 2.10. Statistical Analysis

Statistical analyses for single comparisons were performed by Student's *t*-test, and the differences between the groups were tested using the One Way ANOVA multiple comparisons test using the GraphPad Prims program version 7 *p* < 0.05. All results underwent the Shapiro-Wilk normality test with *p* > 0.05.

## 3. Results

### 3.1. FR3 Inhibits the Gelatinolytic Activity of MMP-2 and MMP-9* In Vitro* and Selectively Decreases the Viability of Tumor Cells

**Figure 1:**
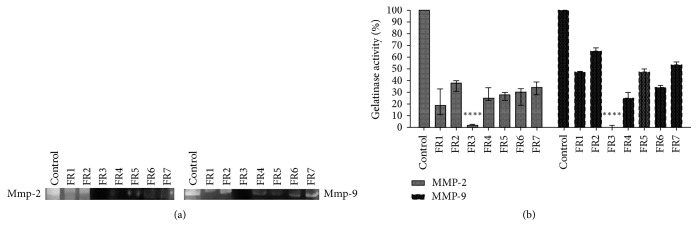
The gelatinolytic activity of MMP-2 and MMP-9 treated with the* B. variegata candida* fractions was inhibited by FR3 (a) when treated for 3 hours of incubation, with a total decrease of the percentage of active gelatinases, significantly different from the other fractions (b). *∗∗∗∗*  represents the significant difference with a value of *p* < 0.0001.

**Figure 2:**
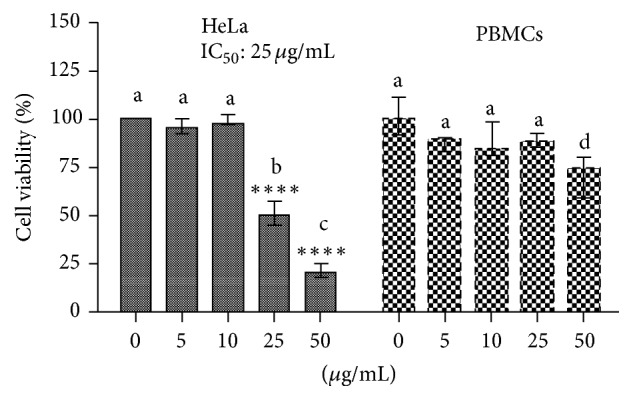
Viability analysis (MTT) of PBMCs and HeLa cell line exposed to the FR3 (0.5; 10; 25; and 50 *μ*g/mL) for 72 hours. The results were expressed in relation to the DMSO control. *∗∗∗∗* indicates statistically significant differences between treatment and control in the same cell line. Different letters indicate statistically significant differences between each treatment between both cell lines (*p* < 0.0001).

We observed that FR3 was the only fraction tested that completely inhibited MMP-2 and MMP-9 (Figure 1), standing out from the others. The FR3 reduced HeLa cell viability in a dose-dependent manner (Figure 2). Furthermore, at higher concentrations (25 and 50 ug/ml) FR3 decreased HeLa cell viability (50.66 ± 6.02% and 21%  ± 3.60, resp.) (IC_50_: 25 *μ*g/mL) and showed lower toxicity to nontumor PBMCs (87.33%  ± 5.03 and 71%  ± 10.81) at the same concentrations.

### 3.2. FR3 Inhibits HeLa Cell Migration and Invasion and the Presence of Active MMP-2 in the Cell Supernatant

**Figure 3:**
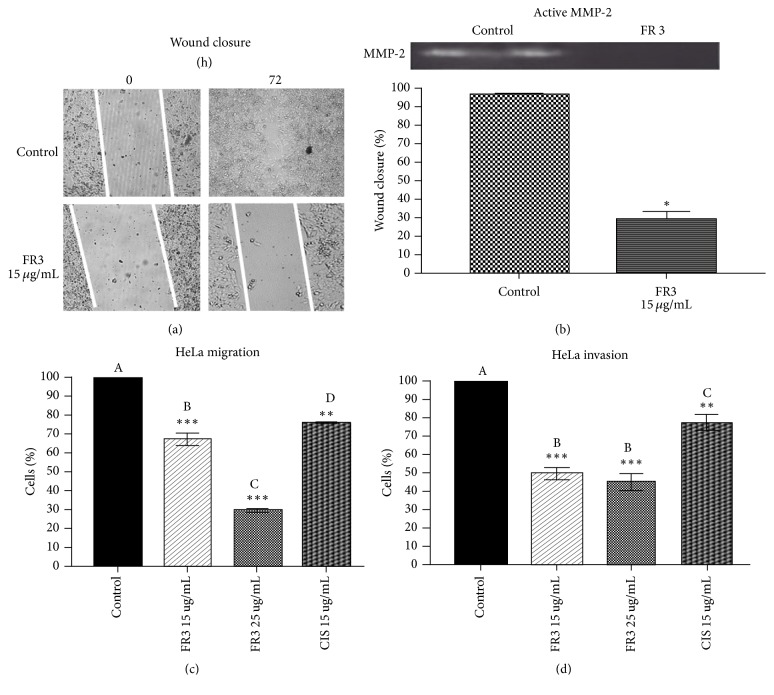
FR3 inhibited wound closure of the HeLa cell monolayer after 72 hours of treatment (a) in 70% (*p* ≤ 0.05) and decreased the expression of active MMP-2 in the supernatant of these cells (b). FR3 inhibited the migration of HeLa cells (15 ug/ml and 30 ug/ml) by the Boyden Chamber assay, as well as treatment with Cisplatin (15 ug/ml) for 24 hours (c) and inhibited the invasion of HeLa cells treated for 24 hours using Matrigel (d). The significant difference with *p* ≤ 0.05 values. *∗∗∗*  indicates statistically significant differences between treatment and control with value of* p* ≤ 0.001. *∗∗*  indicates statistically significant differences between treatment and control with value of* p* ≤ 0.005. *∗*  represents the significant difference with a value of* p* ≤ 0.05. Different letters indicate statistically significant differences between each treatment with* p* ≤ 0.0001.

**Figure 4:**
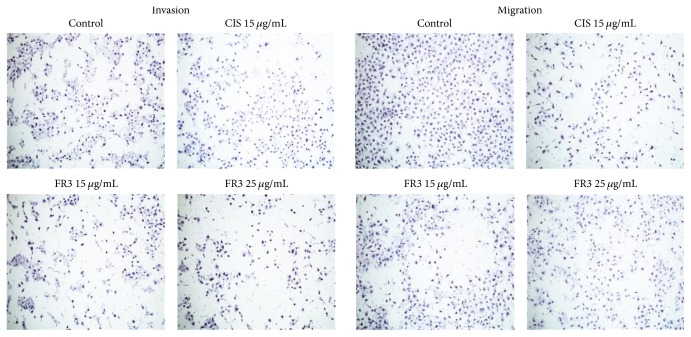
FR3 decreased the number of cells that migrated through the Boyden Chamber in Migration and Invasion assay. 100x to magnification.

FR3 significantly inhibited HeLa cell migration (Figures 3(a) and 3(b)) in the wound closure assay. FR3 showed greater efficacy in HeLa cell transwell migration inhibition than Cisplatin (Figure 3(c)) according to the number of cells counted (Figure 4). Similarly, FR3 inhibited HeLa cell invasion significantly more than Cisplatin in the Matrigel assay (Figure 3(d)) according to the number of cells counted (Figure 4). Active MMP-2 was not observed without supernatant from the treated cells in which the control cell supernatant showed a clear band corresponding to the activity of this enzyme (Figure 3(b)).

### 3.3. FR3 Induces Apoptosis by the Extrinsic Pathway in HeLa Cells

**Figure 5:**
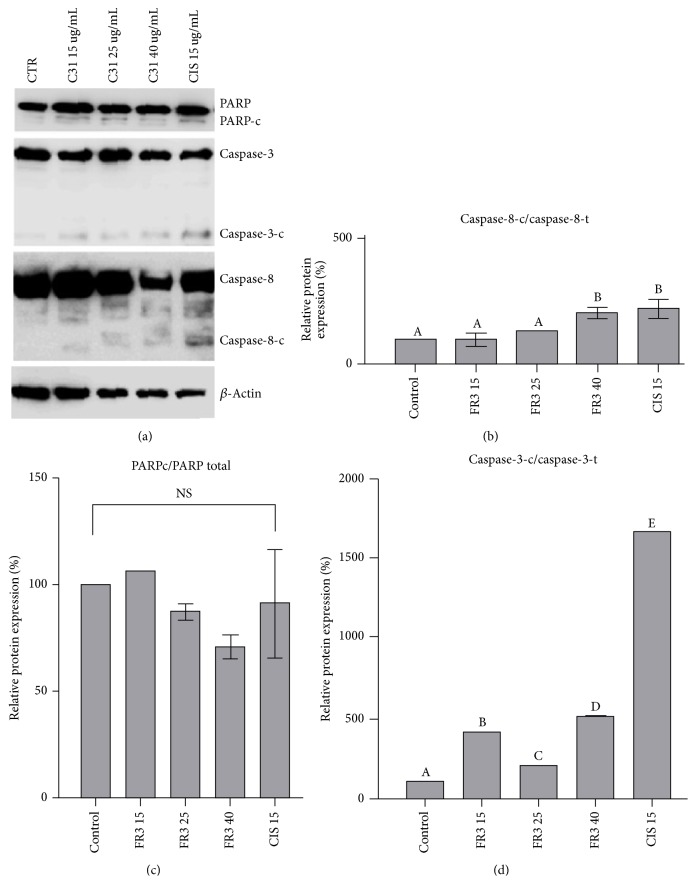
The FR3 acted on PARPc/PARP cell death marker proteins, caspase-3c/caspase-3, and caspase-8c/caspase-8 in 24-hour treated HeLa cells. The concentrations used were 15 *μ*g/mL, 25 *μ*g/mL, and 40 *μ*g/mL FR3 and 15 *μ*g/mL Cisplatin. (b)–(d) Densitometric analysis of the western blot transfer data of the proteins. The significant difference with values of *p* < 0.0001. Different letters indicate statistically significant differences between each treatment with* p* ≤ 0.0001.

HeLa cell treatment with FR3 showed a significant increase (206.2%  ± 20.98, 40 *μ*g/mL) in cleaved caspase-8 expression (Figure 5(b)) and caspase-3c/caspase-3 (15, 25, and 40 *μ*g/mL) (Figure 5(d)). The caspases are a group of cysteine proteases that regulate death mechanisms such as apoptosis.

**Figure 6:**
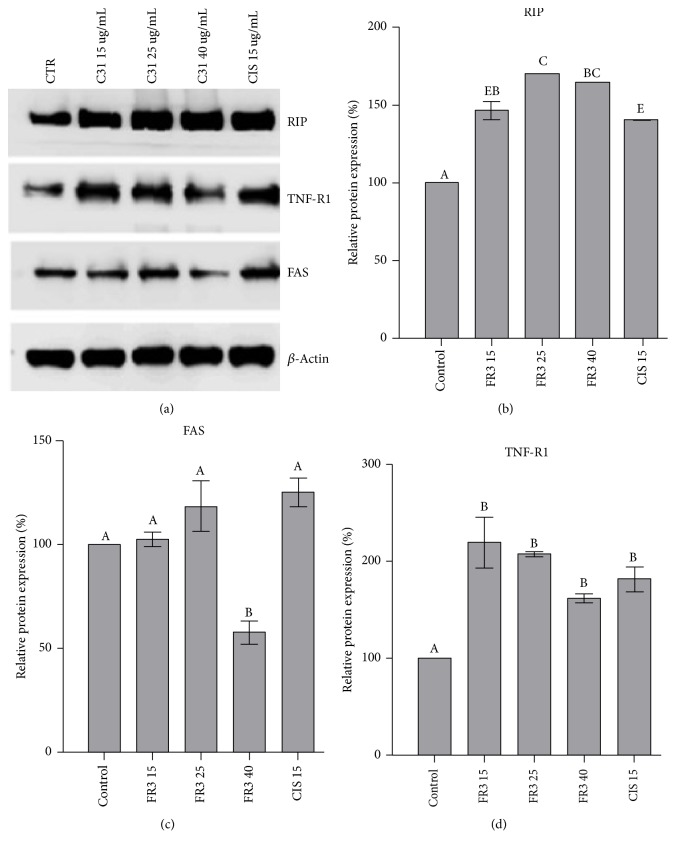
Effect of the FR3 on RIP, FAS, and TNF-R1 in 24-hour treated HeLa cells at concentrations of 15 *μ*g/mL, 25 *μ*g/mL, and 40 *μ*g/mL and Cisplatin at 15 *μ*g/mL concentrations. (b)–(d) Densitometric analysis of the western blot transfer data of the three proteins. The significant difference with values of *p* < 0.0001. Different letters indicate statistically significant differences between each treatment with* p* ≤ 0.0001.

The increase of expression of RIP (a receptor-interacting protein 1, the DD-containing serine/threonine kinase, plays a crucial role in switching between death and survival signaling) and TNF-R1 (canonical death receptor essential for TNF-induced apoptosis) (Figures 6(b) and 6(d)) and decreased FAS expression (40 ug/mL) (Figure 6(c)) are also observed, confirming that FR3 cytotoxicity against HeLa cells was due to induction of cell death by apoptosis. The ratio of PARPc/PARP expression showed no significant difference (Figure 6(c)).

### 3.4. ESI(−)FT-ICR MS Analysis

Among the assigned compounds in FR3 sample using ESI(−)FT-ICR MS, are the ethyl a-D-glucopyranoside, D-pinitol, and fatty acids such as palmitic acid, oleic acid, and phenolic acid. These compounds are present in plant extracts with proven antitumor activities (Table 1).

## 4. Discussion

In the present study, we showed that the* Bauhinia variegata candida* fraction (FR3) inhibits the MMP-2 and MMP-9 gelatinolytic activity. We also demonstrated that this fraction decreased HeLa cell viability and exhibited low toxicity in normal cells. The migration and invasion decreased and the amount of active MMP-2 in the HeLa cell supernatant decreased. Moreover, we showed that exposure to FR3 induced cell death via TNFR-1 and RIP1. Finally, mass spectrometry showed that* Bauhinia variegata candida* fraction FR3 contained glucopyranosides, D-pinitol, fatty acids, and phenolic acid.

It is reported that some plant-derived compounds can exhibit an inhibitory action against MMP-2 and MMP-9 gelatinolytic activity* in vitro* studies [21–23]. However, few studies have interrogated the action of* Bauhinia extracts* with inhibition of these enzymes. In a previous study conducted by our group,* Bauhinia ungulate* extracts also inhibited the activity of MMP-2 and MMP-9 [13]. Thus, it is believed that species of the genus* Bauhinia *may contain promising components with inhibitory action against MMPs. The FR3 presents as a promising compound for cancer treatment, as it was selectively cytotoxic to the HeLa line. It is thought that this activity is correlated with the presence of ethyl a-d-glucopyranoside, already described in* Bauhinia petrandra* extracts [24] and belongs to a class of molecules that has been highlighted by the antitumor activity of this cell line [25, 26]. Another component of FR3 that may be correlated with this activity is palmitic acid, which has selective cytotoxic action on the MOLT-4 leukemia cell line [27]. The FR3 is also considered promising because it inhibits MMP-2 and MMP-9, enzymes that are closely linked to dissemination [28] of a type of cancer considered highly invasive and highly expressed in cervical cancer [29].

Our results showed increased expression of TNFR-1 (tumor necrosis factor receptor), which is a membrane receptor that interacts with FADD proteins (FAS-associated death domain) and which, in turn, recruits caspase-8, which cleaves caspase-3, triggering the cell death process extrinsic pathway. Thus, since FR3 also provided for caspase-8 overexpression, it is possible that the cytotoxicity evidenced in the results is correlated with this cell death pathway [30]. However, intricately, death of HeLa cells may also be related to the newly discovered necroapoptosis pathway, as the results showed overexpression of RIPK1 (receptor-interacting serine/threonine protein kinase 1), which is a protein present in the necrosome, a characteristic structure of necroapoptosis formed from the binding of TNF-*α* to its TNFR-1 ligand on the cell surface [31]. Among the compounds found in FR3, oleic acid, linoleic acid, and glucopyranoside can be correlated with this activity, once they have already demonstrated the activation of the extrinsic pathway of the HeLa lineage [32].

Furthermore, it is believed that the decrease in MMP-2 activity in the cell supernatant is linked to decreased invasion and migration of HeLa cells, as several studies have established a relationship between the* in vitro* antimetastatic potential of plant extracts and the decrease in active MMPs [33–35]. This can be explained by the fact that MMPs act, via the degradation of the extracellular matrix, through mechanisms that allow tumor growth, angiogenesis, and metastasis [36]. D-pinitol, described as a component of FR3, is believed to present some function in this context, as it has already shown ability to inhibit cell migration, wound closure, and invasion of prostate cancer cells [37].

In conclusion, the present study suggests that the molecules present in the FR3 reduce cervical cancer cell viability. Moreover, we demonstrated a reduction on the migration and invasion activity. These finds can be correlated with MMPs inhibitory activity.

## Figures and Tables

**Table 1:** Molecular formulas and compounds estimated from components present in FR3 by ESI(-)M.

Experimental* m*/*z*	Error (ppm)	Proposed compounds	Reference
207.08744	0.11	Ethyl a-D-glucopyranoside	Ndongo et al., 2015
229.04849	0.20	D-Pinitol	Tien-Huang et al., 2013
243.06412	0.13	Ethyl a-D-glucopyranoside	-
255.23301	0.21	Palmitic acid	Harada et al., 2002
281.24867	0.24	Oleic acid	Harada et al., 2002
421.22677	0.5	Phenolic acid	Chen et al., 2012.
